# Relation Classification for Bleeding Events From Electronic Health Records Using Deep Learning Systems: An Empirical Study

**DOI:** 10.2196/27527

**Published:** 2021-07-02

**Authors:** Avijit Mitra, Bhanu Pratap Singh Rawat, David D McManus, Hong Yu

**Affiliations:** 1 College of Information and Computer Sciences University of Massachusetts Amherst Amherst, MA United States; 2 Department of Medicine University of Massachusetts Medical School Worcester, MA United States; 3 Department of Computer Science University of Massachusetts Lowell Lowell, MA United States; 4 Center for Healthcare Organization and Implementation Research Bedford Veterans Affairs Medical Center Bedford, MA United States

**Keywords:** bleeding, relation classification, electronic health records, CNN, GCN, BERT

## Abstract

**Background:**

Accurate detection of bleeding events from electronic health records (EHRs) is crucial for identifying and characterizing different common and serious medical problems. To extract such information from EHRs, it is essential to identify the relations between bleeding events and related clinical entities (eg, bleeding anatomic sites and lab tests). With the advent of natural language processing (NLP) and deep learning (DL)-based techniques, many studies have focused on their applicability for various clinical applications. However, no prior work has utilized DL to extract relations between bleeding events and relevant entities.

**Objective:**

In this study, we aimed to evaluate multiple DL systems on a novel EHR data set for bleeding event–related relation classification.

**Methods:**

We first expert annotated a new data set of 1046 deidentified EHR notes for bleeding events and their attributes. On this data set, we evaluated three state-of-the-art DL architectures for the bleeding event relation classification task, namely, convolutional neural network (CNN), attention-guided graph convolutional network (AGGCN), and Bidirectional Encoder Representations from Transformers (BERT). We used three BERT-based models, namely, BERT pretrained on biomedical data (BioBERT), BioBERT pretrained on clinical text (Bio+Clinical BERT), and BioBERT pretrained on EHR notes (EhrBERT).

**Results:**

Our experiments showed that the BERT-based models significantly outperformed the CNN and AGGCN models. Specifically, BioBERT achieved a macro F1 score of 0.842, outperforming both the AGGCN (macro F1 score, 0.828) and CNN models (macro F1 score, 0.763) by 1.4% (*P*<.001) and 7.9% (*P*<.001), respectively.

**Conclusions:**

In this comprehensive study, we explored and compared different DL systems to classify relations between bleeding events and other medical concepts. On our corpus, BERT-based models outperformed other DL models for identifying the relations of bleeding-related entities. In addition to pretrained contextualized word representation, BERT-based models benefited from the use of target entity representation over traditional sequence representation

## Introduction

### Background

Bleeding refers to the escape of blood from the circulatory system either internally or externally. Bleeding events are common and frequently have a major impact on patient quality of life and survival. Bleeding events are common adverse drug events, particularly among patients with cardiovascular conditions who are prescribed anticoagulant medications [1].

We are seeing a marked increase in the use of anticoagulants, driven predominantly by the increased prevalence of atrial fibrillation (AF), a prothrombotic condition for which anticoagulants are frequently indicated. In the United States, the number of AF patients is increasing rapidly, mostly in the elderly population, with a projection of 12 million by 2050 [2,3]. The chance of having a stroke from AF can be as high as 10% within 5 years of AF diagnosis [4]. Clinicians must weigh stroke risk against the risk of bleeding from anticoagulants [5,6]. Most published data on the risks of anticoagulants come from clinical trials, where major bleeding outcomes are rigorously adjudicated by trained abstractors. However, there are limitations to this approach, as there are many important groups that are underrepresented in clinical trials. Real-world data are lacking, in part owing to the significant time and cost associated with manual chart review, which is the current gold standard for bleeding classification. With a lack of available risk calculators for a situation like this, it is challenging to advise anticoagulants to older AF patients as they are at high risk for both stroke and anticoagulant complications, for example, bleeding [7-9]. Clinicians and researchers would benefit from new ways to classify the relations between bleeding events and related medical entities to provide more accurate risk and benefit assessments of commonly used medications, particularly anticoagulants.

Clinical notes, such as electronic health records (EHRs), contain rich information for various studies including but not limited to epidemiological research, pharmacovigilance, and drug safety surveillance [10,11]. However, bleeding and its attributes are mostly documented in the unstructured EHR narratives instead of the structured fields [10]. With the availability and success of different deep learning (DL) techniques, building accurate and effective DL-based natural language processing (NLP) systems can alleviate this problem and prove viable against more expensive and time-consuming manual annotations. Therefore, in this work, we evaluated different DL models for relation classification between bleeding events and related medical concepts. Relation classification is the task of classifying relations for a pair of target entities from a text span. For example, given the text span “clotted blood was found in the entire colon,” the task is to detect the relation between the bleeding event “clotted blood” and anatomic site “colon.”

A majority of previous studies on clinical text have primarily focused on the relations between medications and other factors such as adverse drug effects (ADEs) [12-15]. However, to our knowledge, there has been no prior work that aims at identifying bleeding event–related relations from EHRs using DL-based NLP systems. The advantages of such systems make them the right group of candidates to investigate for this task.

### Relevant Literature

Realizing the importance of relation classification tasks for clinical narratives, different research groups released several publicly available data sets and launched shared tasks with a focus on relation classification in the clinical domain [15-19]. These include detecting relation types among medical problems, tests, and treatments [16], as well as relations between medications and their various attributes, such as dosage and ADEs [15,17-19]. Our task can be closely compared to any of these tasks.

In general, the relation classification problem can be solved by different systems or models, including rule-based systems, non-DL–based machine learning models, and DL models, depending on the domain and context. For example, Kang et al [20] used the Unified Medical Language System (UMLS) [21] to build a knowledge base where relations between medications and ADEs can be detected based on the shortest path between them. Xu et al [22] applied support vector machines (SVMs) to determine the relation between drugs and diseases, while Henriksson et al [11] used random forest.

Studies have compared non-DL–based machine learning models with DL models for relation classification, and the results are mixed. Munkhdalai et al [12] used a recurrent neural network (RNN) on clinical notes for relation identification and found that an SVM with a rich feature set outperformed the RNN on their data set. In contrast, Luo et al [23] showed that a convolutional neural network (CNN) with pretrained medical word embeddings is superior to traditional machine learning methods. A similar observation was made by He et al [24] for their CNN model with a multipooling operation.

Beyond traditional RNN and CNN models, Li and Yu [13] evaluated a capsule network and multilayer perceptron (MLP) for single domain and multidomain relation classification tasks on EHR data sets and found that although there was a slight improvement, the capsule network model was not superior to the MLP model. Christopoulou et al [14] developed intrasentence models based on bidirectional long short-term memory (bi-LSTM) and attention mechanism. The authors also employed a transformer network [25] for building an intersentence model. For clinical conversations, Du et al [26] proposed a relation span attribute tagging (R-SAT) model that utilizes bi-LSTM and has been shown to outperform the baseline by a large margin for two relation classification tasks.

Recent DL architectures, such as Bidirectional Encoder Representations from Transformers (BERT) [27] and graph convolutional network (GCN), have shown promising results for relation classification across different domains. Wu and He [28] used BERT with entity information for relation classification on the SemEval-2010 Task 8 data set [29] and obtained better results than other state-of-the-art methods. Soares et al [30] introduced a new training scheme for BERT, matching the blank (MTB), which gave superior performance on three different data sets. Lin et al [31] used BERT to solve the sentence-agnostic temporal relation extraction problem for clinical text. Guo et al [32] proposed a novel GCN model with attention and densely connected layers, named the attention-guided graph convolutional network (AGGCN), which utilizes the full dependency tree information of the input sequences. In their experiments, the AGGCN achieved significant performance gain over the other GCN-based systems on multiple relation classification data sets. A GCN has also been employed on different biomedical tasks successfully, including biomedical event extraction [33] and measurement of semantic relatedness between UMLS concepts [34], among others.

Among different DL models, CNN, BERT, and AGGCN are currently the most representative architectures. However, despite being state-of-the-art models, few studies have evaluated the three models parallelly for clinical relation classification, which is the focus of this study.

### Objective

In this study, we focused on the evaluation of three different state-of-the-art DL systems for the relation classification task on a new curated EHR data set. These systems included a CNN, a GCN with attention (AGGCN), and models based on BERT. In particular, a GCN has not yet been explored in any clinical setting for relation classification. The contributions of this work can be summarized as follows: (1) this is the first study to identify the relations between bleeding events and other relevant medical concepts; (2) we provide comparative analyses of three different DL architectures for the relation classification task on a new EHR data set; and (3) we explored the effects of additional domain knowledge on the AGGCN model, as well as how entity position representations influence BERT models’ predictions.

## Methods

### Data Set

With approval from the Institutional Review Board at the University of Massachusetts Medical School and a memorandum of understanding between the University of Massachusetts Medical School and Northwestern University, we annotated 1046 deidentified discharge summaries from patients with cardiovascular diseases who received anticoagulants during their stays at hospitals affiliated with Northwestern University. The notes were annotated by five medical experts under the supervision of two senior physicians. From the comprehensive list of 13 entity types, we chose five relevant to bleeding and the relations among them. This resulted in four relation types for our relation classification study as follows: (1) bleeding event-bleeding anatomic site (Event-Site), (2) bleeding event-bleeding lab evaluation (Event-Lab), (3) bleeding event-suspected alternative cause (Event-AltCause), and (4) bleeding lab evaluation-severity (Lab-Severity).

A *bleeding event* indicates the escape of blood from the circulatory system. Examples of bleeding events from our cohort include mentions such as “hemorrhage,” “black tarry stools,” and “clotted blood.” *Bleeding anatomic site* is the corresponding anatomic site for a bleeding event, for example, “esophagus” in the phrase “blood oozing in esophagus.” *Bleeding lab evaluation* is any relevant laboratory test, and *severity* is the test value when in an abnormal range. *Suspected alternative cause* indicates possible alternative causes for bleeding other than anticoagulants.

Our cohort of 1046 notes included 15,363 relation instances. There was a large variation in token length, ranging from 3 to 985. For our task, we chose a subset that had instances with token length no more than 1000. Since most DL models do not handle long input sequences and 99.11% (15,226) of the 15,363 relation instances had a token length less than 1000, we used these 15,226 instances to build the final data set. This included both intrasentence and intersentence relations. All the relation types and their frequencies for this cohort are provided in Table 1. We also list relation lengths for each relation type, which is the number of tokens between the two target entities. It can be noticed that out of the four relation types on average, Event-Lab and Event-AltCause had significantly longer relation lengths with wider spreads.

**Table 1:** Data statistics.

Relation type	Occurrences	Relation length, mean (SD)
Event-Site	3495	4.81 (10.20)
Event-Lab	3314	93.69 (137.99)
Event-AltCause^a^	4947	48.08 (94.02)
Lab-Severity	3470	3.26 (4.82)

^a^AltCause: suspected alternative cause.

We used the NLTK package [35] to tokenize EHR text. For all experiments, we maintained a train, validation, and test split of 60:20:20 on the note level. We also generated negative relation instances by taking permutations of all possible entity pairs that did not have any relationship between them. For all three splits, this resulted in a set of negative relations that was two to three times the other relations combined. For the training and development sets, we down-sampled the negative relations such that their frequency was similar to the other four relation types combined. We did not perform down-sampling for the test set, so it would be representative of the real EHR note distribution.

**Figure 1:**
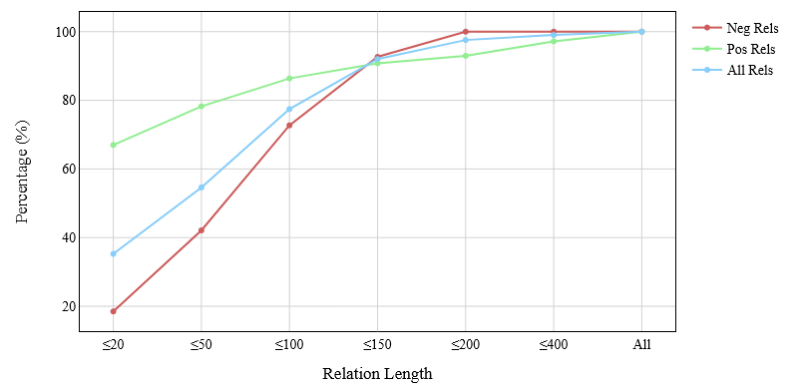
Relation distribution for different relation lengths. Neg: negative; Pos: positive; Rels: relations.

Figure 1 shows the relation distribution in our data set for different relation lengths. The x-axis indicates the range (eg, ≤20 indicates all instances that have a relation length of 20 or lower), and the y-axis indicates the percentage of instances at that range. Positive relations are all relation instances that belong to the four relation types described above. Here, we can see a steep increase for the negative relations compared to the positive relations. This shows that, on average, negative relations had longer relation lengths. For example, as we increased the relation length upper bound from 50 to 100, there was almost a 30% increase in negative relations, whereas for positive relations, it was less than 10%. In particular, negative relations had a mean relation length of 74.01 (SD 49.40). We discuss the implications of relation length in the Results section.

### Models

For this work, we evaluated three different state-of-the-art DL architectures (CNN, GCN, and BERT), which we describe briefly below.

#### CNN

**Figure 2:**
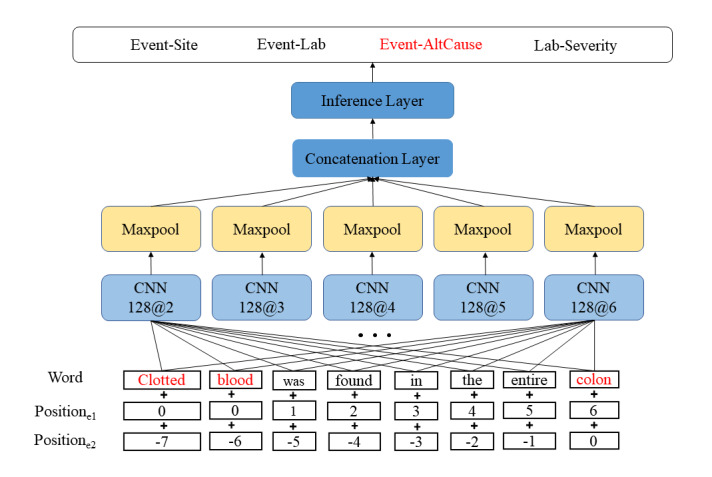
The high-level view of our convolutional neural network (CNN) model. It has five different CNN modules with filters of different window sizes, followed by maxpooling and concatenation. The inference layer includes a dropout, a fully connected layer, and a softmax layer. Positione1 and Positione2 refer to the relative positions of each word from entity1 and entity2, respectively. AltCause: suspected alternative cause.

CNN is a class of deep feed-forward neural networks that is specialized for data with a high degree of temporal or spatial correlation such as image data. CNNs have also been widely used for various NLP tasks with success, including relation classification [36-39]. Our CNN relation classification model was built upon the work of Nguyen and Grishman [37], which is a state-of-the-art CNN architecture for relation classification in the open domain. As shown in Figure 2, the model utilizes five separate convolutional layers with filters of different window sizes to capture rich local n-gram features. For example, “128@2” in the first CNN block indicates 128 filters with a window size of 2. Each layer is followed by a tanh nonlinearity. Finally, we used a maxpool layer, concatenated the output, applied dropout, and added a fully connected layer, followed by a softmax layer for the final classification. As input, we used pretrained word embeddings concatenated with randomly initialized positional embeddings. We used positional embeddings to embed the relative positions of the target entities and other words in a relation instance, as it has been shown to improve various NLP tasks including relation classification [24,40].

#### GCN

**Figure 3:**
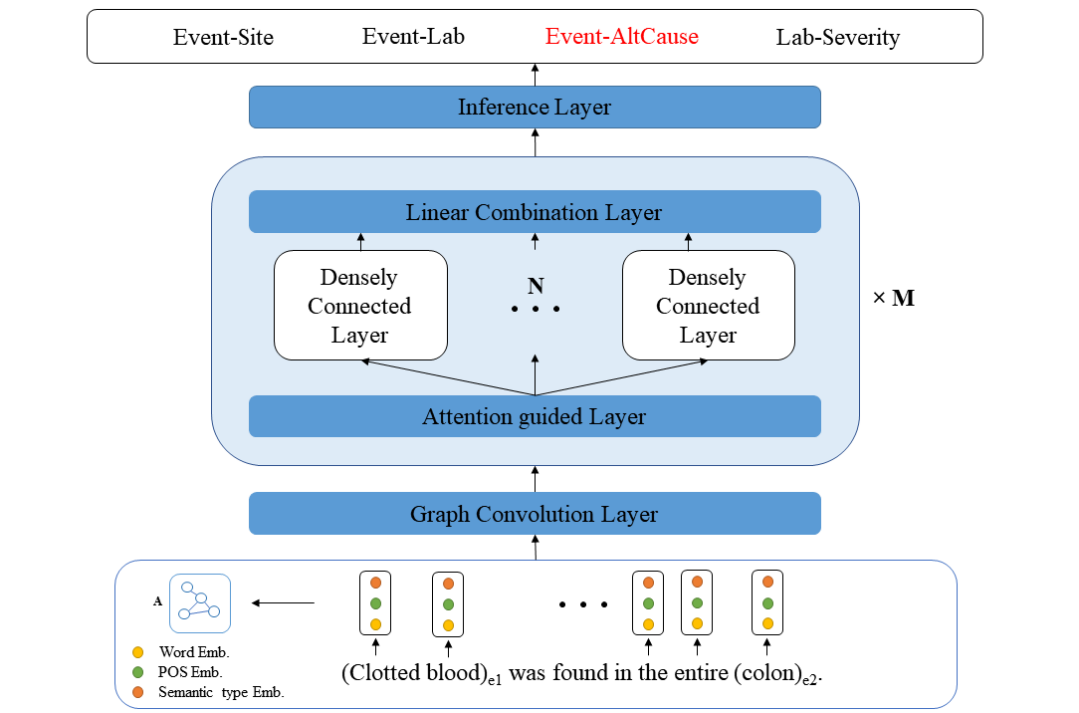
The high-level view of our attention-guided graph convolutional network (AGGCN) model. A is the adjacency matrix used to represent the graph data. The core of the model is comprised of M identical blocks (AGGCN blocks), each with three types of layers as follows: one attention-guided layer, N densely connected layers, and one linear combination layer. Details are available in Multimedia Appendix 1. AltCause: suspected alternative cause; Emb: embedding; POS: parts of speech.

Since semantic coding has enjoyed success in clinical NLP [41], GCNs [42] may be effective and powerful as they represent the semantic or syntactic dependency of input sequences as graphs, which have shown superior performance for the relation classification task in the open domain [32,43]. We implemented the AGGCN [32], which incorporates dense connections for rich dependency information and multihead attention [25] for soft pruning the trees (Figure 3). Here, each sentence corresponds to a graph, represented in the form of an adjacency matrix A, where A_ij_=1 if node *i* and node *j* have an edge between them and A_ij_=0 otherwise. Additional model details are available in Multimedia Appendix 1.

Unlike the previous work [32], we built semantic graphs instead of syntactic graphs. This was motivated by decades of NLP work in the clinical domain that highlights the advantages of semantic parsers [41,44]. To construct the graph, we used the UMLS Metathesaurus [21]. First, we mapped an input sentence to the UMLS concepts using MetaMap [44]. We considered all words in an input sequence as the nodes in a graph, each with a self-loop. Then, for every two nodes, we connected them if they had a semantic relation (eg, child-of) and were identified as at least one of the 26 preselected semantic types. These semantic types were chosen to prioritize bleeding events and relevant entities (Multimedia Appendix 2). However, owing to data sparsity, this resulted in disconnected graphs where most of the nodes had no incoming or outgoing edge. As an alternative, we relaxed the criteria by connecting nodes to each other (belonged to any of the 26 semantic types). In a separate experiment, we repeated the same process with all 127 semantic types from the UMLS Metathesaurus.

In addition, we investigated two different methods, namely, initializing A from a uniform distribution and initializing A with all 1s (all nodes are connected to each other). Finally, we explored semantic-type embeddings (STEs). A comparison of these methods is available in the Results section.

#### BERT

BERT [27] is a language representation model that was pretrained on a large text corpus using unsupervised objectives. BERT has been shown to outperform most of the DL models in various NLP tasks, including clinical applications [45]. At its core, BERT employs bidirectional transformers [25] with multihead attention mechanisms. Paired with an effective pretraining scheme for unsupervised tasks, namely, masked language modeling and next sentence prediction, BERT can provide a rich contextual representation for any text sequence. BERT’s contextualized word representations can be fine-tuned for any downstream NLP task. In this work, we used three variants of BERT (BERT pretrained on biomedical data [BioBERT] [46], BioBERT pretrained on clinical text [Bio+Clinical BERT] [47], and BioBERT pretrained on EHR notes [EhrBERT] [45]), all of which have been shown to improve clinical NLP applications. They all share the same architecture with a difference in their pretraining corpora.

**Figure 4:**
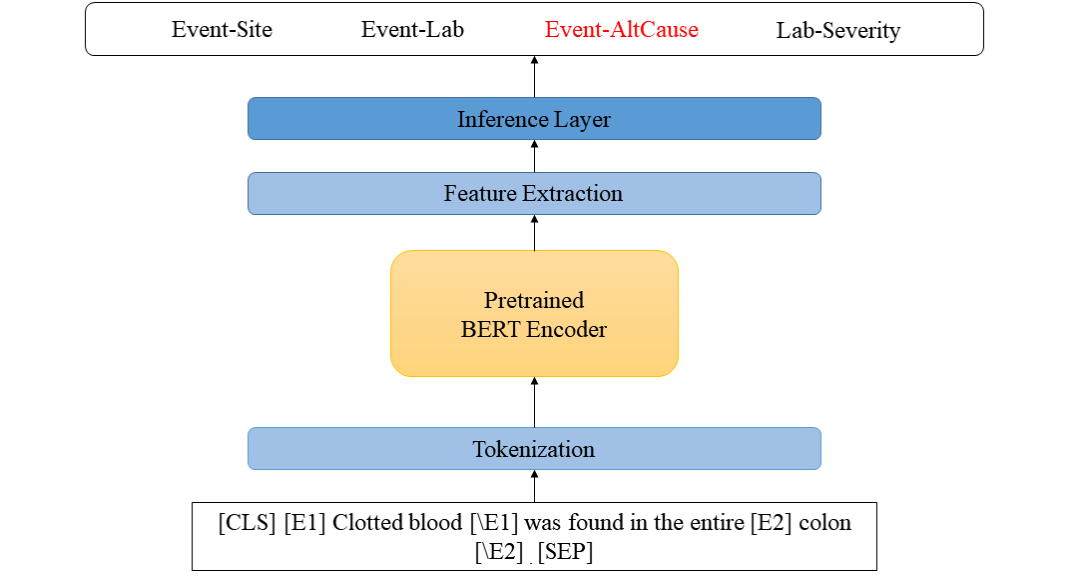
The high-level view of a Bidirectional Encoder Representations from Transformers (BERT)-based model. AltCause: suspected alternative cause.

In our implementation (Figure 4), for a target entity pair, we used four reserved tokens ([E1], [E2], [\E1], and [\E2]) to mark the start and end of the entities. For our task, to handle an input sequence larger than 512 word pieces, we modified the BERT encoder so that it could slide over any input sequence with a stride, essentially splitting the sequence into multiple 512 word piece–long subsequences. It later merges the fine-tuned hidden representations of the subsequences depending on the maximum context window. A maxpool operation is performed over the subsequences’ [CLS] tokens to create the final [CLS] representation. Later the feature extraction module constructs features from the final hidden representations. It can be from either the [CLS] token or a fusion of entity start or end tokens. In particular, we experimented with approaches, such as the maxpool of entity-start tokens ([E1] and [E2]), concatenation of entity-start tokens, and max-pool of entity-end tokens ([\E1] and [\E2]). Details about these are provided in the Results section (Experiments With BioBERT subsection). Finally, we added a fully connected layer on top for the relation classification.

### Evaluation Metrics

All the models were evaluated using precision, recall, and F1 score. We report both micro- and macro-averaged scores. Averaged over all the instances, micro-averaged scores give an overall evaluation and therefore are biased toward the class with the highest instances. On the contrary, macro-averaged scores help obtain a better understanding of the models’ performance across different classes as it is averaged over all the classes.

### Experimental Setup

All model hyperparameters were fine-tuned on the development set. For the CNN model, we included five convolutional layers, each with 128 filters and different window sizes (2, 3, 4, 5, and 6). We chose Adam as the optimizer with a learning rate of 0.01, and the dropout rate was 0.5. The model was trained for 300 epochs. We found 300 and 10 to work the best as the dimensions for word and position embeddings, respectively. For the AGGCN model, we used part-of-speech (POS) embeddings in addition to pretrained word embeddings. Here, the dimensions were 30 and 300, respectively. We ran the AGGCN model for 100 epochs with a learning rate of 0.5 and stochastic gradient descent optimizer. Other hyperparameters included three heads for the attention layer, three AGGCN blocks, two and five sublayers in the first and second dense layers, etc. For both the CNN and AGGCN models, we used global vectors for word representation (GLOVE) [48] as pretrained word embeddings.

We used the popular library Transformers [49] for implementing our BERT models. As mentioned in the Models subsection, we modified the existing implementation so that it could cover sequences of all lengths. We used a stride of 128 with a maximum sequence length of 512. The learning rate was 5×10^-5^ and the dropout rate was 0.1. We initialized each BERT model’s encoder with corresponding pretrained weights. All models were fine-tuned for 15 epochs.

Cross-entropy loss was used for training all the models. In each experiment, we used an early stopping criterion based on the model’s performance on the development set. All models were evaluated on the same hold out test set, and the reported results were averaged over three independent runs. All model trainings and evaluations were performed on Tesla V100 GPUs (Nvidia).

## Results

### Comparison of the Models

We report our results for the relation classification task in Table 2. All BERT-based models did comparatively better than the CNN and AGGCN models. The BioBERT model achieved a 1.3% absolute improvement (*P*<.001) over the AGGCN model in both micro and macro F1 scores, while the difference with the CNN model was even more significant at almost 8% (*P*<.001). A similar performance improvement was observed for the Bio+Clinical BERT model but with a lower recall. The CNN model performed the worst for all relation types. For each model, we also report the macro scores of two ensemble methods (last two rows) where both improved the model performance. *P* values were calculated following the work by Berg-Kirkpatrick et al [50]

**Table 2:** Performance comparison of convolutional neural network (CNN), attention-guided graph convolutional network (AGGCN), and Bidirectional Encoder Representations from Transformers–based models (BERT).

Relation type and performance		Model				
		CNN^a^	AGGCN^b^	BioBERT^c^	Bio+Clinical BERT^d^	EhrBERT^e^
**Event-Site**						
	Precision, mean (SD)	0.910 (0.003)	0.941 (0.009)	0.916 (0.058)	0.929 (0.020)	0.942 (0.024)
	Recall, mean (SD)	0.817 (0.003)	0.947 (0.006)	0.942 (0.009)	0.930 (0.016)	0.920 (0.024)
	F1 score, mean (SD)	0.861 (0.003)	0.944 (0.002)	0.928 (0.027)	0.929 (0.003)	0.977 (0.003)
**Event-Lab**						
	Precision, mean (SD)	0.653 (0.014)	0.619 (0.014)	0.616 (0.029)	0.618 (0.023)	0.587 (0.031)
	Recall, mean (SD)	0.629 (0.011)	0.737 (0.022)	0.793 (0.027)	0.785 (0.010)	0.802 (0.012)
	F1 score, mean (SD)	0.641 (0.003)	0.672 (0.002)	0.692 (0.009)	0.691 (0.011)	0.677 (0.022)
**Event-AltCause^f^**						
	Precision, mean (SD)	0.640 (0.006)	0.718 (0.017)	0.708 (0.048)	0.718 (0.026)	0.721 (0.014)
	Recall, mean (SD)	0.596 (0.012)	0.723 (0.030)	0.828 (0.029)	0.792 (0.015)	0.803 (0.009)
	F1 score, mean (SD)	0.617 (0.004)	0.720 (0.007)	0.761 (0.017)	0.753 (0.008)	0.760 (0.006)
**Lab-Severity**						
	Precision, mean (SD)	0.907 (0.004)	0.967 (0.003)	0.977 (0.005)	0.974 (0.007)	0.963 (0.011)
	Recall, mean (SD)	0.963 (0.001)	0.986 (0.004)	0.993 (0.001)	0.991 (0.001)	0.991 (0.004)
	F1 score, mean (SD)	0.934 (0.002)	0.976 (0.002)	0.985 (0.003)	0.982 (0.004)	0.977 (0.003)
**Micro**						
	Precision, mean (SD)	0.768 (0.006)	0.800 (0.014)	0.786 (0.038)	0.793 (0.020)	0.783 (0.013)
	Recall, mean (SD)	0.739 (0.006)	0.838 (0.015)	0.885 (0.017)	0.868 (0.009)	0.873 (0.005)
	F1 score, mean (SD)	0.753 (0.002)	0.818 (0.001)	0.832 (0.015)	0.829 (0.007)	0.826 (0.009)
**Macro**						
	Precision, mean (SD)	0.777 (0.005)	0.811 (0.010)	0.804 (0.032)	0.810 (0.017)	0.803 (0.007)
	Recall, mean (SD)	0.751 (0.005)	0.848 (0.014)	0.889 (0.016)	0.874 (0.009)	0.879 (0.006)
	F1 score, mean (SD)	0.763 (0.003)	0.828 (0.001)	0.842 (0.012)	0.839 (0.005)	0.836 (0.007)
**Macro (majority voting)**						
	Precision	0.778	0.813	0.822	0.824	0.823
	Recall	0.752	0.849	0.895	0.882	0.887
	F1 score	0.764	0.829	0.855	0.851	0.851
**Macro (averaging predictions)**						
	Precision	0.779	0.813	0.824	0.826	0.828
	Recall	0.753	0.855	0.879	0.879	0.886
	F1 score	0.765	0.833	0.850	0.850	0.854

^a^CNN: convolutional neural network.

^b^AGGCN: attention-guided graph convolutional network.

^c^BioBERT: BERT pretrained on biomedical data.

^d^Bio+Clinical BERT: BioBERT pretrained on clinical text.

^e^EhrBERT: BioBERT pretrained on electronic health record notes.

^f^AltCause: suspected alternative cause.

### Domain Knowledge for the AGGCN

For the AGGCN, we first experimented with different approaches to encode information from graph inputs. The AGGCN uses an *n* × *n* adjacency matrix *A* to represent a graph with *n* nodes. For our inputs, we built the graph based on MetaMap [44], as explained in the Models subsection. To understand the importance of domain-specific knowledge (UMLS), we also removed the UMLS knowledge by connecting all the nodes (tokens) of a graph (input sequence) to each other (all connected). This is equivalent to setting all the elements in *A* to 1. In addition, we also explored a weighted graph (Uniform). For this, we built *A* using a uniform distribution with the half-open interval [0,1).

As shown in Table 3, predefining the graph using the domain knowledge did not improve the overall performance. Several factors may have contributed to this result, including the noise introduced by MetaMap for mapping text to the UMLS concepts and the incompleteness of concept relations in the UMLS. Our results showed that the weighted graph (Uniform) achieved the best performance.

**Table 3:** AGGCN (Attention-guided graph convolutional network) performance with different methods.

Metric and performance		Method^a^				
		MetaMap (26)^b^	MetaMap (All)^c^	All Connected	Uniform	Uniform + STE^d^
**Micro**						
	Precision, mean (SD)	0.774 (0.008)	0.757 (0.026)	0.783 (0.025)	0.800 (0.014)	0.796 (0.011)
	Recall, mean (SD)	0.829 (0.007)	0.852 (0.019)	0.845 (0.018)	0.838 (0.015)	0.836 (0.007)
	F1 score, mean (SD)	0.800 (0.003)	0.801 (0.006)	0.812 (0.005)	0.818 (0.001)	0.816 (0.007)
**Macro**						
	Precision, mean (SD)	0.787 (0.008)	0.781 (0.018)	0.798 (0.019)	0.811 (0.010)	0.805 (0.011)
	Recall, mean (SD)	0.844 (0.007)	0.865 (0.018)	0.855 (0.017)	0.848 (0.014)	0.848 (0.008)
	F1 score, mean (SD)	0.813 (0.003)	0.816 (0.003)	0.824 (0.003)	0.828 (0.001)	0.825 (0.008)

^a^All methods used global vectors for word representation (GLOVE) and part-of-speech (POS) embeddings.

^b^MetaMap (26) used 26 specific semantic types.

^c^MetaMap (All) used all 127 semantic types from the Unified Medical Language System Metathesaurus.

^d^STE: semantic-type embedding.

We also evaluated the effects of STEs. The UMLS had a total of 127 semantic types, from which we identified 26 semantic types relevant to our work (Uniform + STE). For a word with multiple semantic types, we used the semantic type with the highest MetaMap Indexing (MMI) score. Our results with STEs, however, did not improve the performance. We also evaluated POS embeddings and entity-type embeddings. Results from our experiments suggested that only POS embeddings improved performance, while entity-type embeddings slightly degraded performance. Other experiments included the use of different pretrained word embeddings. Surprisingly, we found that the biomedical word embeddings [51] did not perform well compared with the GLOVE embeddings on our data set. In summary, the best combination for AGGCN includes adjacency matrix initialization from uniform distribution and the use of GLOVE and POS embeddings.

### Experiments With BERT

For classification, there are various ways to extract the contextualized sequence representations from BERT. The most common approach is to use [CLS] token embedding. In this work, since entity positions were already encoded in the input sequence, we explored different alternatives [30]. For example, we considered fusing the entity start tokens’ embeddings ([E1] and [E2]) and the entity end tokens’ embeddings ([\E1] and [\E2]). The fusion function was either maxpooling or concatenation. To our knowledge, this is the first study to evaluate different approaches for extracting BERT representation for clinical relation classification.

We used BioBERT as a representative of the BERT-based models, and the results are shown in Table 4. Although [CLS] token embedding is the most common approach, our results suggested that its performance is close to taking the concatenation of the entity start or end tokens’ embeddings. In fact, the best performing method was the maxpool of the entity start tokens’ embeddings, resulting in 1% improvement in the macro F1 score over [CLS]-only representation.

**Table 4:** Effect of different sequence representation methods on the BioBERT (BERT pretrained on biomedical data) model.

Method and performance		Micro	Macro
**[CLS] only**			
	Precision, mean (SD)	0.779 (0.040)	0.803 (0.024)
	Recall, mean (SD)	0.866 (0.015)	0.873 (0.011)
	F1 score, mean (SD)	0.819 (0.015)	0.832 (0.010)
**Maxpool-start tokens**			
	Precision, mean (SD)	0.786 (0.038)	0.804 (0.032)
	Recall, mean (SD)	0.885 (0.017)	0.889 (0.016)
	F1 score, mean (SD)	0.832 (0.015)	0.842 (0.012)
**Maxpool-end tokens**			
	Precision, mean (SD)	0.780 (0.036)	0.800 (0.027)
	Recall, mean (SD)	0.878 (0.015)	0.885 (0.014)
	F1 score, mean (SD)	0.825 (0.014)	0.837 (0.010)
**Maxpool-start tokens + [CLS]**			
	Precision, mean (SD)	0.775 (0.034)	0.794 (0.028)
	Recall, mean (SD)	0.882 (0.014)	0.887 (0.014)
	F1 score, mean (SD)	0.824 (0.014)	0.835 (0.012)
**Concatenate-start tokens**			
	Precision, mean (SD)	0.762 (0.021)	0.787 (0.015)
	Recall, mean (SD)	0.886 (0.011)	0.891 (0.008)
	F1 score, mean (SD)	0.819 (0.008)	0.832 (0.006)
**Concatenate-end tokens**			
	Precision, mean (SD)	0.768 (0.007)	0.793 (0.005)
	Recall, mean (SD)	0.880 (0.009)	0.885 (0.009)
	F1 score, mean (SD)	0.820 (0.005)	0.833 (0.005)
**Concatenate-start tokens + [CLS]**			
	Precision, mean (SD)	0.743 (0.034)	0.777 (0.021)
	Recall, mean (SD)	0.895 (0.008)	0.898 (0.006)
	F1 score, mean (SD)	0.811 (0.017)	0.827 (0.013)

### Effect of Relation Length

**Figure 5:**
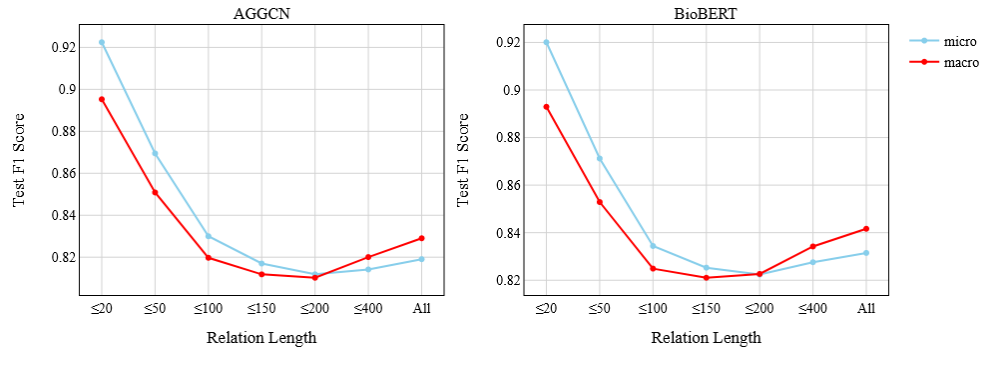
Effect of relation length on model performance. The x-axis indicates the subset range, for example, "≤20" indicates the test subset that consists of all the instances with a relation length of 20 or lower. AGGCN: attention-guided graph convolutional network; BERT: Bidirectional Encoder Representation from Transformers; BioBERT: BERT pretrained on biomedical data.

As pointed out in Table 1, the four relation types have a wide range of relation lengths. Relation length (ie, the number of words between the target entities) acts as context and hence can influence the training process. To demonstrate how it affected our trained models, we created multiple subsets of our test set, each with a different range for relation length. Each subset contained only those test instances that had a relation length within the subset range. We chose the AGGCN and BioBERT models and ran inference on all the test subsets. The results are shown in Figure 5.

For both models, the test F1 scores kept decreasing until the relation length range reached 200, with an exception for the BioBERT macro score that had the lowest F1 score at 150. After this point, the macro F1 scores surpassed their respective micro scores, and surprisingly, both models’ F1 scores improved despite the increase in relation length. This is slightly counterintuitive, as a larger relation length should have been difficult for the models to understand. To understand this behavior, we manually reviewed the gold labels and model predictions for all the test instances that had a relation length of 200 or higher. As expected, we found that all these instances were from either the relation types (Event-Lab and Event-AltCause) or a negative relation. Interestingly, all the model predictions were also within these three types. This shows that the models learned the correlation between relation length and relation type as a shortcut [52] and consequently did not consider Event-Site and Lab-Severity as possible relation types for longer relation lengths, resulting in improved overall performance. Our analyses showed the limitations of machine learning models in that they might learn from correlations, not causality, and this might lead to model overfitting.

However, a drawback of learning this shortcut is labeling many negative relations as Event-Lab or Event-AltCause, as negative relations have long relation lengths on average (refer to the Dataset subsection). For both models, this generated many false positives, resulting in low precision. This also explains the huge difference between precision and recall for these two relation types (Table 2).

### Model Performance With Data Size

**Figure 6:**
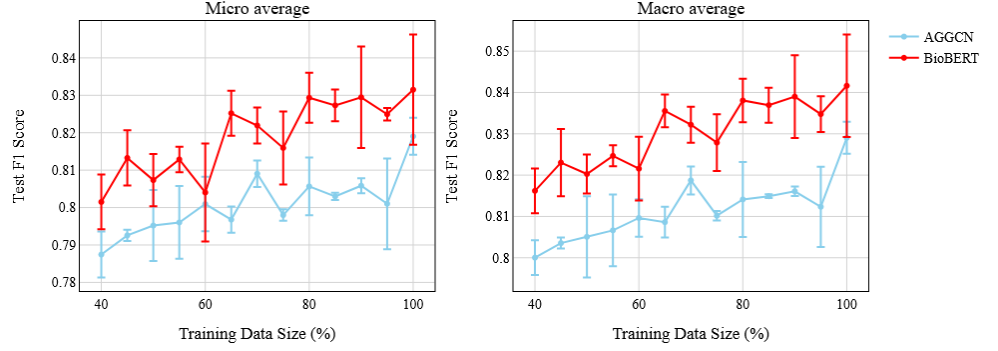
Effect of training data size on model performance. Each error bar indicates the standard deviation range at the corresponding point. AGGCN: attention-guided graph convolutional network; BERT: Bidirectional Encoder Representation from Transformers; BioBERT: BERT pretrained on biomedical data.

For any supervised DL method, the amount of available labeled data almost always plays a key role in the overall model performance. In our task, we wanted to evaluate how this affects the models, namely AGGCN and BioBERT. To this end, we trained both models with different portions of the training data separately and measured their performances. We observed an upward trend (Figure 6) for both, indicating that more training data would be better for our clinical relation classification task irrespective of the model type and metric averaging criterion. However, the AGGCN appeared to have less deviation (low standard deviation) with more data (a high slope), as opposed to BioBERT, for which the deviations were higher, although the performance differences remained statistically significant between the two models.

## Discussion

### Principal Findings

The results of our experiments demonstrated that fine-tuned BERT-based models outperformed both the CNN and AGGCN models by a significant margin. This can be attributed to the richer and contextualized representation of the pretrained BERT models compared to pretrained word embeddings, such as GLOVE, even when paired with POS embeddings and domain knowledge (for AGGCN). In our experiment, we found that the CNN significantly underperformed the AGGCN and BERT-based models by a large margin, primarily because of its inability to capture the global context of the input sequences. On the other hand, although all BERT-based models outperformed the AGGCN model by relatively small margins, they were statistically significant (*P*<.001).

Despite model architectural differences, all models had better performance on the Event-Site and Lab-Severity relation types (eg, F1 scores of 0.928 and 0.985, respectively, for BioBERT). However, their performances for Event-Lab and Event-AltCause were relatively poor (eg, F1 scores of 0.692 and 0.761, respectively, for BioBERT). As shown in Table 1, these two relation types had comparatively larger relation lengths. This phenomenon would result in difficulty in annotation, thereby negatively impacting performance. Moreover, the lengthy context could pose challenges for the DL models as well. Both could have contributed to the overall poor performance for these two categories. In addition, except for the CNN model, we observed significant differences between precision and recall.

Our results showed that incorporating the concept relations from the UMLS did not improve AGGCN’s performance. One possible reason might be the data sparsity, that is, only few concepts were connected in the graph input for the AGGCN. When a token is not identified by MetaMap as relevant but is important for classifying the instance, putting a 0 in its corresponding node position in the adjacency matrix *A* sends an erroneous signal to the model. This is a possible area for improvement, and we will work on this as part of our future work. On the other hand, *A* initialized with a uniform distribution gave the best recall and a better F1 score. This approach might seem counterintuitive as it does not necessarily pass any useful information unlike a dependency tree. However, this can be reasoned as the input dependency tree serves as an initialization, helping the attention-guided layers to build multiple edge-weighted graphs. This acts as a soft-pruning strategy where the model learns how the nodes should be connected to each other and on which connections to focus.

A quick look at the standard deviations reveals that Bio+Clinical BERT and EhrBERT were more stable than BioBERT, as both had utilized large scale EHR notes for the pretraining process. BioBERT had the highest F1 score, but different instantiations of the network gave widely different results, contributing to the higher standard deviation. The AGGCN was also better than BioBERT in this regard. Thus, we suggest using Bio+Clinical BERT or EhrBERT when stability is the primary concern. BioBERT on the other hand had the highest recall, which may be an important criterion for clinical applications. For the AGGCN, the key advantage was the model being lightweight and consequently having a faster inference (Multimedia Appendix 3).

### Error Analysis

We conducted error analysis for the two relations (Event-Lab and Event-AltCause) where models performed poorly for both recall and precision scores. We analyzed the BioBERT model and made the following observations:

1. Most incorrect predictions were false positives, driven by the target entity types. For example, the model incorrectly predicted an Event-Lab relation in “Irrigation catheter was placed in ED and [hematuria]_e1_ has improved. Repeat [H&H]_e2_ is >8 and bleeding has stopped.”

2. Another common source of error was the model incorrectly labeling a negative relation sequence that described a patient’s medical history that was not directly related to the present diagnosis. For example, “Likely source thought to be upper GIB given hx of bleeding [ulcer]_e1_ in past + [hematemesis]_e2_.” Here, the model predicts the relation Event-AltCause between the target entities. Though the entity *GIB* can be a suspected alternative cause, both target entities are from the patient’s previous history.

3. Another reason for error was the existence of the relation in the instance but between different entities. For example, take the negative relation instance “Daily CBC show anemia ([Hbg]_e1_ 8.7 - 8.8, current at 8.7), with low Fe, transferrin+TIBC wnl, high ferritin. Labs support hemolytic anemia with low haptoglobin, high LDH, high tbili and indirect bili. Per inpatient attending read, blood smear showed no schistocytes, bite cells or heinz bodies, with few reticulocytes visualized per hpf, final report pending. CT kidney/pelvis showed no gross GU abnormalities and left gluteal [hematoma]_e2_.” Here, the model predicted an Event-Lab relation though *Hbg* and *hematoma* do not have any such relation. However, there is an Event-Lab relation here between *Hbg* and *anemia*.

4. Limited corpus size and no additional domain knowledge made it difficult for the model to make predictions on relation instances with never-observed words or medical acronyms. In some cases, it was worsened due to the lack of grammatical consistency and coherent patterns.

### Conclusions

In this work, we studied three state-of-the-art DL architectures for a relation classification task on a novel EHR data set. Our work is the first to identify the relations between a bleeding event and related clinical concepts. Our results showed that BERT-based models performed better than attention-guided GCN and CNN models. Further experiments suggested that semantic graphs built using the UMLS semantic types and relations between them did not help the GCN model. On the other hand, incorporating entity token information improved the performance of BERT-based models. We also demonstrated the impacts of relation length and training data size. In our future work, we plan to explore richer domain knowledge and distant supervision. Additionally, leveraging our earlier work on named entity recognition (NER) [53], we aim to build a joint learning pipeline that integrates both NER and relation classification for bleeding events and relevant medical concepts.

## Appendix

Multimedia Appendix 1 Attention-guided graph convolutional network (AGGCN).

Multimedia Appendix 2 Relevant semantic types from the Unified Medical Language System.

Multimedia Appendix 3 Training time and parameter size.

## Abbreviations

ADE: adverse drug effect
AF: atrial fibrillation
AGGCN: attention-guided graph convolutional network
AltCause: suspected alternative cause
BERT: Bidirectional Encoder Representations from Transformers
bi-LSTM: bidirectional long short-term memory
Bio+Clinical BERT: BioBERT pretrained on clinical text
BioBERT: BERT pretrained on biomedical data
CNN: convolutional neural network
DL: deep learning
EHR: electronic health record
EhrBERT: BioBERT pretrained on EHR notes
GCN: graph convolutional network
GLOVE: global vectors for word representation
MLP: multilayer perceptron
NER: named entity recognition
NLP: natural language processing
POS: part-of-speech
RNN: recurrent neural network
STE: semantic-type embedding
SVM: support vector machine
UMLS: Unified Medical Language System
