# Supplementary material for: Relation Classification for Bleeding Events From Electronic Health Records Using Deep Learning Systems: An Empirical Study
Source: JMIR Med Inform. 2021 Jul 2;9(7):e27527. doi: 10.2196/27527 (PMC8285744; doi:10.2196/27527)
Supplement: Multimedia Appendix 1 [file medinform_v9i7e27527_app1.docx]

**Multimedia Appendix 1.** Attention-guided graph convolutional network (AGGCN).

The AGGCN model is composed of an embedding layer, a graph convolution network, *M* AGGCN blocks and a relation classification network. An AGGCN block is formed from an attention guided layer, *N* densely connected layers and a linear combination layer. Both *M* and *N* are model hyperparameters. The principal components are as follows.

### Graph Convolutional Network (GCN)

A graph with *n* nodes can be represented as an *n × n* adjacency matrix **A**, where **A***_ij_* = 1 if node *i* and node *j* have an edge between them, 0 otherwise. Also, a self-loop is added for each node. For an input feature representation of **h**^(^*^l-1^*^)^ the convolution operation can be expressed as:

$$\mathbf{h}_{i}^{(l)} = RELU(\sum_{j=1}^{n} \mathbf{A}_{ij}\mathbf{W}^{(l)}\mathbf{h}_{j}^{(l-1)}+\mathbf{b}^{(l)})$$

where $\mathbf{h}_{i}^{(l)}$ is the output for node *i* at the *l*-th layer, **W**^(^*^l^*^)^ Is the weight matrix, **b**^(^*^l^*^)^ is the bias vector and RELU is the activation function. The initial input $h_{i}^{(0)}=x_{i}$ is a *d*-dimensional feature vector which can be the pre-trained word embedding concatenated with other embeddings (POS, semantic type etc.) or the output of a bi-LSTM layer for the word representing node *i.*

### Attention Guided Layer

This layer transforms the original graph into a fully connected graph with weighted edges. This is accomplished by generating an *n × n* attention-guided adjacency matrix **Ã** where **Ã***_ij_* denotes the weight of the edge that goes from node *I* to node *j*. **Ã** is computed using multi-head attention [25]. For head *t* at layer *l*,

$$\mathbf{Ã}^{(t)} = softmax(\frac{Q\mathbf{W}_{i}^{Q}\times(K\mathbf{W}_{i}^{K})^{T}}{\surd d})V$$

where both query *Q* and key *K* are the feature representation **h**^(l-1)^ from the previous layer, and $\mathbf{W}_{i}^{Q}\in\mathbb{R}^{d\times d}$ , $\mathbf{W}_{i}^{K}\in\mathbb{R}^{d\times d}$ are the respective weight matrices for *Q* and *K*. The total number of heads, *N* is a hyperparameter for the model.

### Densely Connected Layer

Each **Ã*^t^*** from the attention guided layer is followed by a densely connected layer. Dense connections [51] are added to capture the local and global dependencies among the nodes. This enables training a deeper model more efficient and effective on a larger input graph. For any node *j*, at layer *l*, the concatenation of the previous *(l-1)* layers can be expressed as $\mathbf{g}_{j}^{(l)}$ which can be mathematically defined as,

$$\mathbf{g}_{j}^{(l)} = \boldsymbol{[}\mathbf{h}_{j}^{(0)};\mathbf{h}_{j}^{(1)};...;\mathbf{h}_{j}^{(l-1)}]$$

Each densely connected layer has multiple sub-layers, *L* which is another hyperparameter for the model architecture. Each sublayer has a dimension of $d_{hidden} = d/L$*.* The model requires *N* densely connected layers as there are *N* attention guided adjacency matrices generated from the attention guided layer. For head *t* at layer *l*, this results in the following computation,

$$\mathbf{h}_{t_{i}}^{(l)} = RELU(\sum_{j=1}^{n} \mathbf{Ã}_{ij}^{(t)}\mathbf{W}_{t}^{(l)}\mathbf{g}_{j}^{(l-1)}+\mathbf{b}_{t}^{(l)})$$

Here $\mathbf{W}_{t}^{(l)}\in\mathbb{R}^{d_{hidden}\times d^{(l)}}$ is the weight matrix where $d^{(l)}= d+d_{hidden}\times(l-1)$and $\mathbf{b}_{t}^{(l)}$ is the bias vector.

### Linear Combination Layer

To combine the N densely connected layers’ outputs, the AGGCN model utilizes a linear combination layer defined as:

$$\mathbf{h}_{comb} = \mathbf{W}_{comb}\mathbf{h}_{out}+\mathbf{b}_{comb}$$

where, $\mathbf{h}_{out} = \boldsymbol{[}\mathbf{h}^{(1)};...;\mathbf{h}^{(N)}]\in\mathbb{R}^{d\times N}$ , $\mathbf{W}_{comb}\in\mathbb{R}^{(d\times N)\times d}$ is the weight matrix and **b***_comb_*  is the bias vector.

### Inference Layer

The final relation classification is performed at two stages, first, the sentence representation *h_sent,_* and the target entity representations *h_e1_* and *h_e2_* are computed. *h_sent_* can be computed the following way:

$$h_{sent} = f(f_{mask}(h_{comb}))$$

The above equation takes the output from the linear combination layer as input, uses a mask to remove padded tokens’ feature representation through function *f_mask_* and then performs a maxpooling operation over the sequence length *n* via $f : \mathbb{R}^{d\times n}\mathbb{\to R}^{d\times1}$. The entity representations are obtained in the similar fashion:

$$h_{e_{i}} = f(f_{e\_mask}(h_{comb}))$$

where *f_e_mask_* masks out all the entity representations except the target entity and *i = {1,2}* for the two target entities. Later, all three representations are concatenated and passed to two fully connected (FC) layers. The output from the last FC layer is fed to a log softmax function for obtaining the probability distribution across all the relation types.
