# Supplementary material for: Relation Classification for Bleeding Events From Electronic Health Records Using Deep Learning Systems: An Empirical Study
Source: JMIR Med Inform. 2021 Jul 2;9(7):e27527. doi: 10.2196/27527 (PMC8285744; doi:10.2196/27527)
Supplement: Multimedia Appendix 2 [file medinform_v9i7e27527_app2.docx]

**Multimedia Appendix 2.** Relevant semantic types from the Unified Medical Language System.

For our task, we chose the following 26 semantic types from the UMLS Metathesaurus - Amino Acid, Peptide, or Protein; Acquired Abnormality; Antibiotic; Biologically Active Substance; Body Substance; Body system; Body, Part, Organ or Organ Component; Cell or molecular Dysfunction; Clinical Attribute; Diagnostic Procedure; Disease or syndrome; Experimental model of disease; Finding; Health Care Activity; Injury or Poisoning; Laboratory procedure; Laboratory or Test result; Organ or Tissue function; Pathologic function; Physiologic function; Pharmacologic substance; Qualitative Concept; Quantitative Concept; Spatial Concept; Sign or symptom and Therapeutic or preventive procedure. These semantic types were carefully chosen to cover all the gold label entities from our corpus.
