# Supplementary material for: Relation Classification for Bleeding Events From Electronic Health Records Using Deep Learning Systems: An Empirical Study
Source: JMIR Med Inform. 2021 Jul 2;9(7):e27527. doi: 10.2196/27527 (PMC8285744; doi:10.2196/27527)
Supplement: Multimedia Appendix 3 [file medinform_v9i7e27527_app3.docx]

**Multimedia Appendix 3.** Training time and parameter size.

We measured the training time and total number of trainable model parameters for AGGCN and BioBERT. We found that AGGCN is significantly lighter with 8 million trainable parameters as opposed to 109 million for BioBERT. Being a lighter network, AGGCN also enjoys a faster training time with an average of 4 minutes and 35 seconds per training epoch on a single GPU. BioBERT, on the other hand, took 23 minutes and 12 seconds on average for a single epoch on the same GPU. Consequently, AGGCN also has a faster inference time. Despite being slightly outperformed by BioBERT, all these make AGGCN more suitable for fast prototyping and real-time deployment.
